# Mirror Neuron Activity During Audiovisual Appreciation of Opera Performance

**DOI:** 10.3389/fpsyg.2020.563031

**Published:** 2021-01-27

**Authors:** Shoji Tanaka

**Affiliations:** Department of Information and Communication Sciences, Sophia University, Tokyo, Japan

**Keywords:** action, alpha, aria, EEG, emotion, gamma, mirror neuron, music

## Abstract

Opera is a performing art in which music plays the leading role, and the acting of singers has a synergistic effect with the music. The mirror neuron system represents the neurophysiological mechanism underlying the coupling of perception and action. Mirror neuron activity is modulated by the appropriateness of actions and clarity of intentions, as well as emotional expression and aesthetic values. Therefore, it would be reasonable to assume that an opera performance induces mirror neuron activity in the audience so that the performer effectively shares an *embodied performance* with the audience. However, it is uncertain which aspect of opera performance induces mirror neuron activity. It is hypothesized that although auditory stimuli could induce mirror neuron activity, audiovisual perception of stage performance is the primary inducer of mirror neuron activity. To test this hypothesis, this study sought to correlate opera performance with brain activity as measured by electroencephalography (EEG) in singers while watching an opera performance with sounds or while listening to an aria without visual stimulus. We detected mirror neuron activity by observing that the EEG power in the alpha frequency band (8–13 Hz) was selectively decreased in the frontal-central-parietal area when watching an opera performance. In the auditory condition, however, the alpha-band power did not change relative to the resting condition. This study illustrates that the audiovisual perception of an opera performance engages the mirror neuron system in its audience.

## Introduction

Opera is a performing art that employs the synergistic effect of music and acting, in which music plays the leading role. Singing and acting are under the metacognitive control of the performers (Concina, 2019). Expert musicians have a high level of metacognitive control skills, which include self-control and monitoring. Metacognitive competence is a key factor for self-evaluating one’s performance (Hart, 2014). The performance plays a critical role in expressing operatic emotions in the way that the performer had intended. Two components of opera performance, i.e., singing and acting, influence emotions in the audience primarily through visual and auditory perception. In terms of emotional and aesthetic responses, consistency between the visual perception of the stage performance and the auditory perception of singing may be a critical factor in intracerebral processing. However, to date, how visual and auditory perception modulates such processing remains unknown. It is therefore worthwhile to individually evaluate the effects of singing and acting, alongside their synergism.

According to previous studies in neuroscience, bodily expressions are translated into internal representations in observers by mirror neuron activation (Gallese et al., 2011; Rizzolatti and Fogassi, 2014). Mirror neurons are active both during self-performances and while watching others performing similar actions. The mirror neuron system “mirrors” the behaviors of others, such that the observer feels as if they are performing the actions themselves. Neuroimaging studies in humans have isolated the mirror neuron network, whose principal nodes consist of the ventral premotor cortex and inferior parietal lobule (Calvo-Merino et al., 2005; Iacoboni and Dapretto, 2006). Mirroring is not a passive visual response to actions; instead, it is considered to play important roles in social cognition, such as understanding the intentions of others’ actions, as well as emotional communication between the performer and observer (Montgomery et al., 2007; Rizzolatti and Fogassi, 2014). Facial expressions, for example, contain both motor and emotional components. Further inclusion of the insula and amygdala in the mirror neuron system (van der Gaag et al., 2007) points to the contribution of the mirror neuron system to the comprehension of facially expressed emotions in addition to the motor component of facial expressions. Therefore, the mirror neuron system is a neuronal substrate by which action representation and emotions are shared between the performer and observer.

Mirror neuron activity is detected using neuroimaging methods, such as functional magnetic resonance imaging (fMRI) and electroencephalography (EEG). While fMRI has a superior spatial resolution, high noise, and strong bodily constraints make it inadequate for experiments using music. EEG recordings, however, are quiet and bodily constraints are minimal, which are advantageous in experiments using music. In EEG studies, “mu suppression” or the suppression of alpha oscillations (8–13 Hz) over the brain’s sensorimotor region has been used as an indicator for detecting mirror neuron activity (Pineda, 2005; Fox et al., 2016; Hobson and Bishop, 2016; Wu et al., 2016; Bowman et al., 2017). Mu suppression was discovered by Gastaut and Bert (1954), and a recent meta-analysis concluded that mu suppression provides a valid means for the study of human mirror neuron activity (Fox et al., 2016). There is, however, a notion that the suppression of alpha oscillations in general confounds with several factors that are irrelevant to mirror neuron activity: attentional demands alter the amplitudes of alpha oscillations while suppression of alpha oscillations is also observed during visual processing, which occurs in the occipital regions (Bacigalupo and Luck, 2019). Source identification of mirror neuron activity is limited in studies employing EEG; however, studies with simultaneous EEG and fMRI measurements reported that mu suppression correlated with the blood-oxygen-level-dependent (BOLD) signals in the brain regions consisting of the mirror neuron network, including the dorsal premotor cortex, primary somatosensory cortex, and inferior parietal lobule (Arnstein et al., 2011; Hobson and Bishop, 2017).

One of the notable features of mirror neuron activity is its dependence on *experience*. Mirror neuron activity is stronger when the observed action has been previously experienced by the observer (Cannon et al., 2014; Brunsdon et al., 2020). In their EEG study, the authors compared alpha-band powers during the observation of a relatively novel action of tool handling among three groups of participants: those trained to perform this action (performers), those with only the experience of observing the action (observers), and those who were novices. The performers group exhibited the greatest alpha (mu) suppression. Previous studies suggested that dance performance induces mirror neuron activity in dancers. Compared with a visual baseline condition, the EEG power in the alpha and lower beta frequency bands (7.5–25 Hz) was significantly reduced in dancers, but not in non-dancers, while the participants were watching dance movements (Orgs et al., 2008). Poikonen et al. (2018a; 2018b) analyzed phase synchrony between EEG signals recorded while dancers, musicians, and non-experts were watching dance performance. The phase synchrony in the theta band over the frontal regions was increased in dancers, whereas alpha synchrony was decreased in all groups, suggesting that cortical communication was enhanced in dancers. Taken together, these results suggest that mirror neuron activity reflects the activation of acquired neuronal representations of actions.

Another notable feature of mirror neuron activity is its *multimodality*. Mirror neurons are also responsive to auditory stimuli, the sounds associated with actions (Aziz-Zadeh et al., 2004; Gazzola et al., 2006). For example, the sounds of piano melodies activate the mirror neuron network in pianists (Bangert et al., 2006; Wu et al., 2016). However, studies by Jäncke and coworkers showed an overall increase in EEG power during music listening for all frequency bands (Jäncke et al., 2015, 2018; Markovic et al., 2017), suggesting that no mirror neuron activity was elicited. The authors interpreted this result as an indication of increased internal attention so that the participants were “drawn into the music.” In any event, behind these phenomena, underlies the multisensory integration of auditory and visual stimuli. An fMRI study demonstrated that a combination of watching a choreographed dance and listening to its music together enhanced the activity in the posterior superior temporal sulcus, a multisensory area, as revealed by conjunction analysis (“audiovisual vs. auditory” and “audiovisual vs. visual”) (Jola et al., 2013). Another study reported that watching an opera performance increased emotional arousal and valence compared with only listening (Balteş et al., 2011). The emotional highlights in an opera may be products of multimodal information processing in the brain. Therefore, cautious consideration is needed when one studies opera performance, which is multimodal in nature.

Considering the previous findings, it is reasonable to assume that opera performance induces mirror neuron activity in the audience. However, the individual contributions of visual perception of acting performance and auditory perception of singing to mirror neuron activity remains unknown. Since mu suppression was found to occur when pianists passively listened to piano melodies (Wu et al., 2016), it is possible that the auditory appreciation of an opera aria elicits mirror neuron activity in the audience. Otherwise, we can conclude that opera performance elicits mirror neuron activity through audiovisual perception. To test this hypothesis, this study analyzed EEG signals when participants watched video-clips of opera scenes with sounds, when listened without visual stimulus, and when at rest.

## Materials and Methods

### Participants

Twenty-one right-handed healthy Japanese individuals (19 women and two men, age range: 25–50 years, mean age: 33.6 years) participated in this study. All participants were vocalists in classical music who performed in operas and concerts. None of the participants had any current or past neurological or psychiatric illnesses. This study was approved by the ethics committee of Sophia University, and all participants provided written informed consent prior to participation in the study. The participants were required to be familiar with operas, understand the lyrics, and have experience with performing onstage. This study did not include non-musicians.

### Tasks

The tasks employed in this study were audiovisual, auditory (listening), and resting sessions. Participants watched video-clips of opera scenes with the accompanying sounds, projected on a screen in front of them. Each participant chose an opera scene with an aria from her/his repertoires of opera performance. Participants were asked to choose, if possible, opera scenes from those we had pre-selected. Selected opera scenes were “Donde lieta usci” from *La Bohème* by G. Puccini (six sopranos), “Quando me’n vò” from *La Bohème* by G. Puccini (five sopranos), “Va! Laisse couler mes larmes” from *Werther* by J. Massenet (five mezzo-sopranos), “Porgi, amor, qualche ristoro” from *Le nozze di Figaro* by W.A. Mozart (one soprano), “Deh! tu, bell’anima” from *I Capuleti e i Montecchi* by V. Bellini (one mezzo-soprano), “Habanera” from *Carmen* by G. Bizet (one mezzo-soprano), “Quando le sere al placido” from *Luisa Miller* by G. Verdi (one tenor), and “Per me giunto” from *Don Carlo* by G. Verdi (one baritone). The participants also listened to the same videos in the absence of visual stimulus by turning off the projector. EEG recordings were taken while watching the videos with sounds, listening to the videos without visual stimuli, and, as a control, resting. EEG recordings were made for the whole sessions in the audiovisual and auditory conditions, which ranged from 2 min 10 s to 3 min 30 s, depending on the scenes. The recording time for the resting session was 60 s. In both the listening and resting sessions, participants kept their eyes open so as to be in a condition similar to that of the audiovisual task, thereby excluding the effects of closing the eyes. During all tasks, the participants were sitting on a chair and keeping still without particular movements. The participants were instructed to enjoy the scene or the aria as an audience. Evaluation of the opera performance by the participants was not required.

### EEG Recording

Scalp EEG signals were recorded using a wearable headset with 32 active dry-type gold alloy EEG electrodes (g.Nautilus, Guger Technologies or g.tec Medical Engineering GmbH., Austria), placed according to the International 10–20 System. Continuous EEG signals were sampled at 250 Hz and filtered with a 0.1–100 Hz bandpass filter. A notch filter was also used to suppress 50 Hz power-line interference. A headset with dry electrodes was mounted on the heads of the participants. We used a small, medium, or large-sized cap that fit each participant’s head. The active dry electrodes, g.Sahara, have eight pins, thereby increasing the contact surface area. Because of the round tip of pins, the participants reported a good fit without pain. Prior studies have demonstrated no significant differences in the basic characteristics of recorded EEG signals, such as waveforms and power spectra, between wet and dry electrodes (Fiedler et al., 2015; Grummett et al., 2015; Melnik et al., 2017). During the recordings, the participants were asked to be relaxed and still without particular movements.

### Preprocessing

Electroencephalography signals were preprocessed using EEGLAB version 14.1.2 (Delorme and Makeig, 2004). An independent component decomposition was performed with EEGLAB’s *runica* algorithm. Thirty-two components were decomposed in all participants. Components contaminated with artifacts related to eye movements and blinks were removed, and the remaining ones were back-reconstructed to EEG signals. Preprocessed data were subjected to the frequency and coherence analyses described in Section “Analysis.”

### Analysis

#### Band Powers

The band powers or the average powers for the four frequency bands (theta: 4–8 Hz, alpha: 8–13 Hz, beta: 13–30 Hz, and gamma: 30–45 Hz) of EEG signals in each channel were calculated using MATLAB version R2018a Update 6 (Natick, MA, United States: The MathWorks Inc.). The calculation was made by integrating the power spectral density over the corresponding frequency ranges.

#### Detection of Mirror Neuron Activity

The tentative criterion for detecting mirror neuron activity is to observe “mu suppression” or the suppression of alpha oscillations (8–13 Hz) in the sensorimotor region of the brain (Fox et al., 2016; Hobson and Bishop, 2016; Wu et al., 2016; Bowman et al., 2017). The alpha-band powers at individual electrodes, calculated in “Band Powers”, were subjected to paired *t*-tests embedded in MATLAB, to determine whether there was a statistical difference between the audiovisual and resting conditions, as well as between the listening and resting conditions. For multiple testing, a false discovery rate (FDR) corrected *p*-value of 0.05 was considered significant. The effect size was estimated by calculating Cohen’s *d*.

## Results

**FIGURE 1:**
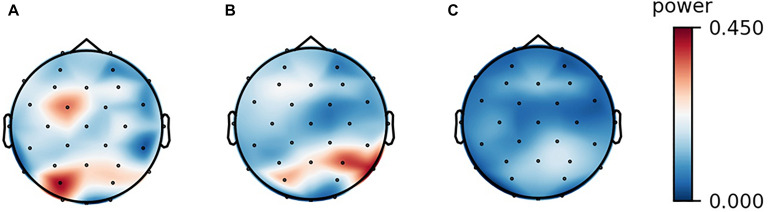
Scalp maps of the alpha-band power of a participant in the three experimental conditions. **(A)** resting, **(B)** listening, and **(C)** watching with sounds.

**FIGURE 2:**
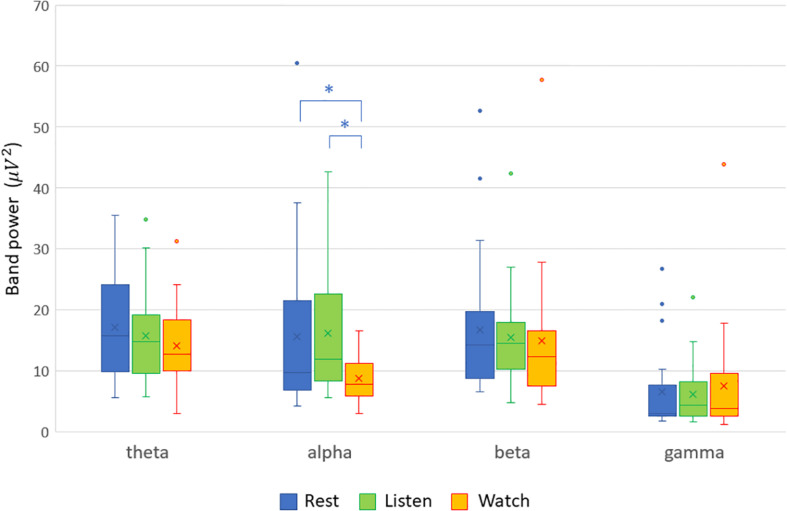
Band powers at the central electrode site (Cz) in the three experimental conditions (resting, listening, and watching with sounds). The alpha-band powers in the audiovisual (Cohen’s *d* = −0.640; *p* = 0.0079) and auditory conditions (Cohen’s *d* = −0.774; *p* = 0.0019) were significantly lower than that in the resting condition, indicated with asterisk (^∗^).

The analyses of the recorded EEG signals yielded the following results. Among the band powers for the four frequency bands (theta, alpha, beta, and gamma), only the alpha-band power was significantly lower in the audiovisual condition than in the resting condition mainly in the frontal, central, and parietal scalp locations. In the auditory condition, the alpha-band power did not show any significant difference at any of the electrode sites. These results are demonstrated by the scalp maps of the alpha-band power in the three experimental conditions (Figure 1). The four band powers at the central electrode site (Cz) in the three experimental conditions are shown in Figure 2. The alpha-band powers in the audiovisual and auditory conditions were significantly lower than that in the resting condition.

**FIGURE 3:**
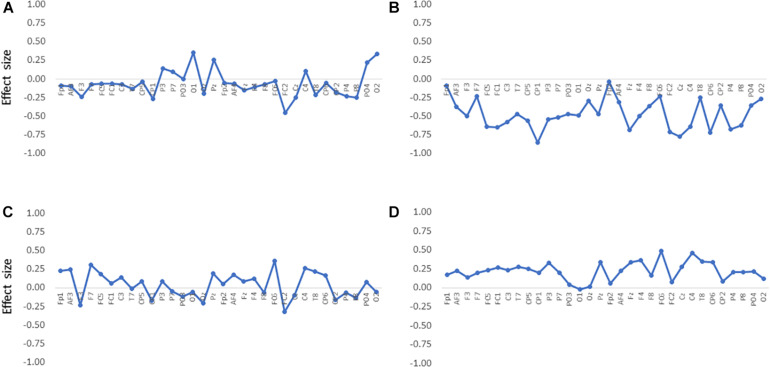
Differences in band powers between the audiovisual condition and the auditory condition at all electrodes in the **(A)** theta, **(B)** alpha, **(C)** beta, and **(D)** gamma bands. The effect size was estimated by calculating Cohen’s *d*.

**FIGURE 4:**
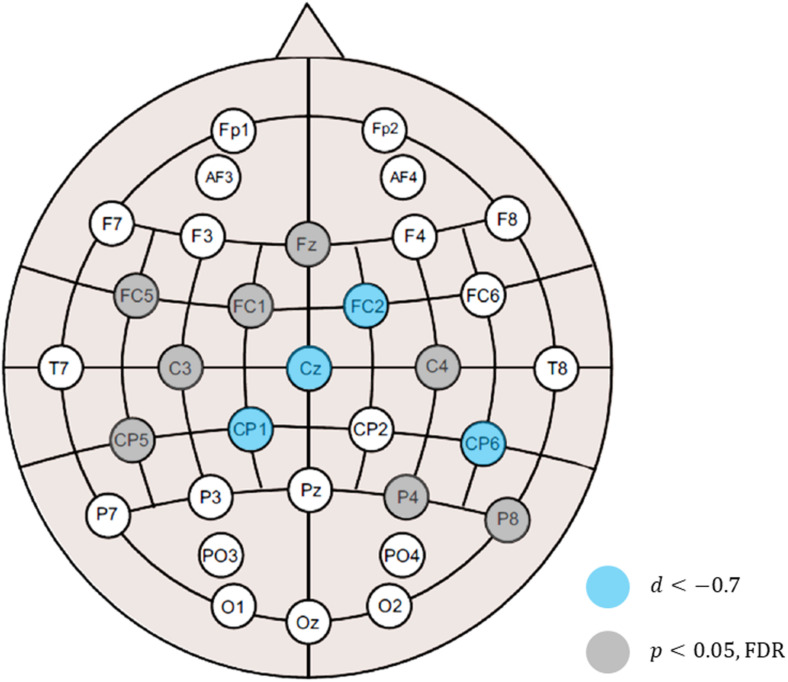
Electrode locations at which the alpha-band power was significantly lowered in the audiovisual condition relative to the auditory condition. Statistical significance after multiple testing correction was observed at 12 electrodes, including 4 electrodes with relatively large effect sizes at FC2 (*d* = –0.72; *p* = 0.027, FDR), Cz (*d* = –0.78; *p* = 0.027, FDR), CP1 (*d* = –0.86; *p* = 0.027, FDR), and CP6 (*d* = –0.72; *p* = 0.027, FDR).

When analyzing alpha suppression, the selection of the control condition or baseline is a matter of debate because alpha oscillations, in general, are sensitive to the attentional state (Bazanova and Vernon, 2014; Hobson and Bishop, 2017; Pagnotta et al., 2020). In this study, contrary to the resting session, which did not require any attentional demands, the auditory and audiovisual sessions made the participants attentive to stimuli. Therefore, we also analyzed the difference in the band powers between the auditory and audiovisual conditions. The differences at all the electrodes are shown in Figure 3, where it can be observed that the alpha-band powers in the audiovisual condition were lower than in the auditory condition. This figure also shows that the gamma-band powers in the audiovisual condition were slightly higher than those in the auditory condition. However, the differences were not statistically significant. Figure 4 shows the electrode locations at which the alpha-band power significantly differed between the audiovisual and auditory conditions. The alpha-band power was significantly lower in the audiovisual condition at 12 electrodes, including 4 electrodes with relatively large effect sizes at FC2 (*d* = −0.72; *p* = 0.027, FDR), Cz (*d* = −0.78; *p* = 0.027, FDR), CP1 (*d* = −0.86; *p* = 0.027, FDR), and CP6 (*d* = −0.72; *p* = 0.027, FDR). The electrodes where the alpha-band power was significantly lower in the audiovisual condition, including these four electrodes, were distributed over frontal-central-parietal regions.

## Discussion

### Alpha Frequency Band

The EEG signals analyzed in this study showed characteristic changes in the alpha frequency band during audiovisual appreciation of an opera performance. The analysis revealed marked differences in the band power between the audiovisual vs. auditory (listening) conditions. The alpha-band power was significantly decreased in the audiovisual condition relative to the resting and auditory conditions. However, only listening to an aria did not significantly change the alpha-band power compared with the resting condition.

### Mirroring Opera Performance

Among the band powers for the four frequency bands (theta, alpha, beta, and gamma), only the alpha-band power was decreased in the audiovisual condition relative to the resting and auditory conditions. Since the auditory condition did not decrease alpha-band power, visual input would be crucial to this decrement. However, because the alpha-band power of EEG signals in the occipital region was not decreased during the audiovisual condition, this decrement is not attributable to visual perception *per se*. Moreover, the comparison of band powers between the auditory and audiovisual conditions revealed significantly lower alpha-band power in the audiovisual condition. A decrease was observed in frontal-central-parietal regions of the scalp, making it likely, therefore, that the alpha suppression observed in this study reflects mirror neuron activity associated with audiovisual integration. Notably, the present study also observed an increase of gamma-band power in the audiovisual condition at a trend level of significance. Previous studies have suggested an association between gamma oscillations and multisensory integration (Friese et al., 2016; Misselhorn et al., 2019), and that alpha oscillations coupled with gamma oscillations mediate selective attention and perceptual binding (Foxe and Snyder, 2011; Zhang et al., 2019). As such, audiovisual perception enables the mirroring of the opera performance in the observer.

### Auditory Appreciation

This study demonstrated alpha (mu) suppression in the audiovisual condition but not in the auditory condition. In previous studies, however, auditory mirror neuron activity has been reported. For example, when pianists passively listened to piano melodies, auditory stimuli were able to activate the mirror neuron network when sounds were linked to actions (Wu et al., 2016). Even a simple auditory or motor task activated the mirror neuron network in pianists, but not in non-musicians (Bangert et al., 2006). In an fMRI study, listening to musical rhythms activated the supplementary motor area, premotor cortex, and cerebellum (Chen et al., 2008), consistent with mirror neuron activation, and generally, mirror neurons can be activated by a stimulus in the auditory domain (Aziz-Zadeh et al., 2004; Lahav et al., 2007; Chapin et al., 2010). These results, however, raise a question as to why the auditory appreciation of an aria in this study did not elicit alpha (mu) suppression. In the auditory condition, vocalists listened to arias. Unlike musical instrumentalists, such as pianists and violinists, vocalists have their own “instruments” in their bodies, and the vocalization method differs individually. The acoustic properties are unique to their instruments. Whereas instrumentalists readily associate sounds of an instrument with its manipulation, it may be unlikely that listening to an aria would be easily associated with an apparent action. Therefore, we infer that listening to an aria did not induce detectable mirror neuron activity but evoked a different intracerebral process.

### Scene Construction

During listening to an aria, the participants might have mentally constructed a “scene” by recalling a memory modified by the auditory perception of an aria. Scene construction is an *internal* process (Hassabis and Maguire, 2007), distinct from mirror neuron activity. In a study by Jäncke et al. (2015), the EEG powers in all frequency bands examined (theta, alpha, and beta) were increased while participants were listening to a well-known aria, leading to the suggestion that this state was characterized by increased internal attention. Whilst alpha-band power decreases when participants pay attention to an external stimulus, it *increases* when participants focus on internal consciousness (Magosso et al., 2019). The notion of scene construction is consistent with these results and the results of a recent study on decision-making in soccer (Fink et al., 2018). In the latter study, participants imagined themselves as acting players and were prompted to imagine a move that could lead to scoring a goal. The alpha-band power of their EEG signals was decreased at the parietal and occipital electrode sites. However, the decrease was weaker when the decisions involved a higher creative potential than when the opposite was involved. The authors suggested that the participants who generated moves that were more creative were more intensely engaged in motor imagery, which relatively increased the alpha-band power. The internal process, consisting of constructing motor imagery, had an opposite effect on alpha-band power to that of mirror neuron activity. Therefore, the present study’s results suggest that the auditory appreciation of an aria led the participants to imagine the opera scene. Note, however, that this result does not exclude the possibility that the auditory perception elicits mirror neuron activity. Since scene construction and mirror neuron activity have opposing effects, as suggested by reciprocal functional connectivity (Tanaka and Kirino, 2019), the reduction in alpha-band power reflecting mirror neuron activity might have been canceled out by the effect of the internal scene construction.

### Performance Evaluation

The reality or subjectivity in enacting a role may be a prerequisite for the engagement of mirror neuron activity. Mirror neuron activity allows for the matching of the internal motor representation and the perceived visual images of actions (Engel et al., 2008; Ishida et al., 2015). Furthermore, it has been demonstrated that mirror neuron activity is modulated by emotional information processing, such as facial expressions (Moore and Franz, 2017; Karakale et al., 2019) and music (McGarry et al., 2014), and possibly by the appropriateness (Caggiano et al., 2009), and clarity of intention of the actions (McGarry et al., 2014). On the other hand, there was a positive correlation between empathy self-reporting and mirror neuron activity (Baird et al., 2011). It is therefore likely that mirror neuron activity is modulated by the degree to which the observer feels the vividness and emotional expression of the action. Emotional expression in an opera needs to be realistic for the audience to interpret them as natural. Otherwise, the audience would not feel any empathy toward the characters on stage. These features of mirror neuron activity can provide useful information to evaluate opera performance. Therefore, this study proposes that analysis of mirror neuron activity during watching an opera performance is useful for its evaluation.

### Limitations

The detection of mirror neuron activity relied on assessing alpha (mu) suppression in this study. The finding that the alpha-band power was significantly lowered at the frontal-central-parietal scalp locations in the audiovisual condition compared with the auditory condition is supportive of its worth. However, this result is not direct evidence of mirror neuron activity. For more convincing results, comparable with those in fMRI studies, more systematic analyses, including source localization and network analysis, with a larger sample are needed.

During the audiovisual appreciation of an opera scene, audiovisual integration was anticipated to occur. Since the gamma band power was modestly increased in the audiovisual condition compared with the auditory condition, we consider that this increase is attributable to audiovisual integration. To obtain evidence of such integration, it would be theoretically conceivable to include a third condition in which the participants watch an opera scene without sounds to compare the result with those in the auditory and audiovisual conditions. However, unlike dance watching (Jola et al., 2013), watching a scene of aria singing without sounds may not produce a meaningful response, a point yet to be clarified. Therefore, employing more systematic analyses instead might be more helpful to draw a firm conclusion.

Participants were required to be familiar with operas, understand the lyrics, and have experience with performing on stage, and the opera scenes were chosen from their repertoires of opera performance. As a consequence, the chosen scenes differed across participants. Although the analyses in this study were restricted to a small group, the variability of the auditory and visual properties of individual scenes might have affected the results. Standardization of stimuli is still a challenge for future studies. For the same reason, this study did not employ non-musicians. Investigating how non-musicians respond to opera appreciation is another topic of research, which is beyond the scope of the present study. It would be meaningful to compare the responses between experts and non-musicians in a future study.

When a performance elicits mirror neuron activity in the audience, it is also of interest to see how the performance is represented in the performer’s brain. However, performance usually accompanies bodily movements. Extracting and analyzing EEG data are challenging because EEG data recorded during movement are mixed with a large number of electromyographic signals. By analyzing EEG data with movement signals, a previous study extracted the expressive components of movements from the recorded EEG data (Cruz-Garza et al., 2014). If feasible, analysis of EEG signals simultaneously recorded from a performer and an audience will provide useful information for the objective performance evaluation.

## Conclusion

The major findings in this study are summarized as follows. First, opera appreciation induced mirror neuron activity in the participants, and audiovisual perception of the performance played a critical role in the mirroring. The neuronal processing for audiovisual perception is *externally* oriented. Second, different conditions of exposure, audiovisual vs. auditory, induced distinct neural processing in the participants. Auditory appreciation without visual stimulus did not seem to induce mirror neuron activity in the audience. Participants might have enjoyed mental scene construction while listening to an aria. This neuronal processing is *internally* oriented. Lastly, all the participants in this study were singers who had experience with opera performance. Mirror neuron activity is modulated by several factors linked to the performance, such as the level of realism, emotional expression, and aesthetic values. Therefore, performance-related features extracted from EEG data could be used as an objective indicator of opera stage performance.

## Data Availability Statement

The datasets generated for this study are available on request to the corresponding author.

## Ethics Statement

The studies involving human participants were reviewed and approved by the Ethics Committee of Sophia University. The participants provided their written informed consent to participate in this study.

## Author Contributions

The author confirms being the sole contributor of this work and has approved it for publication.

## Conflict of Interest

The author declares that the research was conducted in the absence of any commercial or financial relationships that could be construed as a potential conflict of interest.
