# Corrigendum: Mirror neuron activity during audiovisual appreciation of opera performance

**DOI:** 10.3389/fpsyg.2023.1329662

**Published:** 2023-11-21

**Authors:** Shoji Tanaka

**Affiliations:** Department of Information and Communication Sciences, Sophia University, Tokyo, Japan

**Keywords:** action, alpha, aria, EEG, emotion, gamma, mirror neuron, music

In the original article, there was an incorrect description in the caption of Figure 2. The second sentence previously stated, “The alpha-band powers in the audiovisual (Cohen's *d* = −0.640; *p* = 0.0079) and auditory conditions (Cohen's *d* = −0.774; *p* = 0.0019) were significantly lower than that in the resting condition, indicated with asterisk (^*^).” This should instead be written as:

The alpha-band power in the audiovisual condition was significantly lower than that in the resting condition (Cohen's *d* = −0.640, *p* = 0.0079) and that in the auditory condition (Cohen's *d* = −0.774, *p* = 0.0019), indicated with asterisk (^*^).

The full corrected caption for Figure 2 is below.

Figure 2. Band powers at the central electrode site (Cz) in the three experimental conditions (resting, listening, and watching with sounds). The alpha-band power in the audiovisual condition was significantly lower than that in the resting condition (Cohen's *d* = −0.640, *p* = 0.0079) and that in the auditory condition (Cohen's *d* = −0.774, *p* = 0.0019), indicated with asterisk (^*^).

The authors apologize for these errors and state that this does not change the scientific conclusions of the article in any way.

The original article has been updated.

